# Effects of Common Fig (*Ficus carica*) Leaf Extracts on Sperm Parameters and Testis of Mice Intoxicated with Formaldehyde

**DOI:** 10.1155/2016/2539127

**Published:** 2016-01-19

**Authors:** Majid Naghdi, Maryam Maghbool, Morteza Seifalah-Zade, Maryam Mahaldashtian, Zohreh Makoolati, Seyed Amin Kouhpayeh, Afsaneh Ghasemi, Narges Fereydouni

**Affiliations:** ^1^Department of Anatomical Sciences, Medicine School, Fasa University of Medical Sciences, Fasa 7461686688, Iran; ^2^Department of Pathology, Faculty of Medicine, Fasa University of Medical Sciences, Fasa 7461686688, Iran; ^3^Department of Animal Biosystematics, Shahid Beheshti University, Tehran, Iran; ^4^Department of Pharmacology, Faculty of Medicine, Fasa University of Medical Sciences, Fasa 7461686688, Iran; ^5^Department of Health Education and Promotion, School of Public Health, Fasa University of Medical Sciences, Fasa 7461686688, Iran

## Abstract

Formaldehyde (FA) is the leading cause of cellular injury and oxidative damage in testis that is one of the main infertility causes. There has been an increasing evidence of herbal remedies use in male infertility treatment. This assay examines the role of* Ficus carica* (Fc) leaf extracts in sperm parameters and testis of mice intoxicated with FA. Twenty-five adult male mice were randomly divided into control; sham; FA-treated (10 mg/kg twice per day); Fc-treated (200 mg/kg); and FA + Fc-treated groups. Cauda epididymal spermatozoa were analyzed for viability, count, and motility. Testes were weighed and gonadosomatic index (GSI) was calculated. Also, histoarchitecture of seminiferous tubules was assessed in the Haematoxylin and Eosin stained paraffin sections. The findings showed that FA significantly decreased GSI and increased percentage of immotile sperm compared with control group. Disorganized and vacuolated seminiferous epithelium, spermatogenic arrest, and lumen filled with immature germ cells were also observed in the testes. However, Fc leaf extracts improved sperm count, nonprogressive motility of spermatozoa, and GSI in FA-treated testes. Moreover, seminiferous tubule with spermatogenic arrest was rarely seen, indicating that Fc has the positive effects on testis and epididymal sperm parameters exposed with FA.

## 1. Introduction

Infertility is one of the major health problems of reproductive-age couples [1] with increasing incidence rates in males [2]. It is reported that sperm quality has decreased during the 20th century [3]. The cause of this is unknown but is theorized to be due to the number of chemical pollutants in the environment [4]. Recently, several studies have reported the effects of exposure to occupational chemicals on semen quality [5]. Formaldehyde (FA, H_2_CO), one of the simplest organic molecules, is an important chemical that has a wide use in science, households, and industry [6, 7]. The harmful effects of FA are well documented for the respiratory and hematological systems [8–10]. Also, the negative impacts of FA on the reproductive system and sperm parameters were investigated in several reports [7, 10–20]. Some studies showed that FA usage in mice can lead to testicular atrophy and decrease in diameter of seminiferous tubules, height of seminiferous epithelial cells, and testes weight [7, 10, 17]. It disrupts the Leydig cells and inhibits the steroidogenesis in mouse testes [13, 14]. FA exposure can also decrease the motility and number of spermatozoa [17], induce apoptosis of spermatogenetic cells, and inhibit spermatogenesis in testicular tissue [11, 15, 18–20]. It was shown that FA is the leading cause of cellular injury and oxidative damage in many tissues through increasing the production of reactive oxygen species (ROS) [11].

Nowadays, antioxidants are widely used to break the oxidative chain reaction [21–24]. Recently, herbal medicines may be the preferred choice in male infertility treatment. Probably, the presence of antioxidant in the plants was the main reason behind their activity against infertility [25]. One of the herbs that is widely used for this purpose is common fig (*Ficus carica*, Fc), from the family Moraceae, native to the Middle East and western Asia [26]. Some studies have described the presence of several antioxidant compounds in the leaves, pulps, and peels of this herb [27–34]. It was shown that Fc leaves have the strongest antioxidant potential relative to pulps and peels of this herb, explained by the highest amounts of phenolic compounds occurring in leaves [27] that have the ability to scavenge free radicals, chelate prooxidant metal-ions, and inhibit some enzymes [35, 36]. Samsulrizal et al. observed the positive effects of* Ficus deltoidea* on the sperm motility, count, and testosterone level in diabetic rats. In their study, administration of* Ficus deltoidea* caused positive changes in maintaining healthy sperm parameters [37].

Little is known on the use of herbal medicines by infertile patients, especially men with sperm parameters problems. The current study is an additional documentation on reproductive ethnopharmacology of traditionally used Iranian medicinal herb Fc leaf extracts in mice intoxicated with FA.

## 2. Material and Methods

### 2.1. Preparation of Hydroalcoholic Extracts of Fc Leaf

The leaves of Fc were collected from the end of spring until the beginning of autumn, from Shiraz Province Botanical garden, South of Iran, and authenticated and deposited in the Herbarium of the Fasa University with the voucher specimen number of 100-2. The leaves were washed with distilled water, dried, and blended; 10 g leaf powder was dissolved in 125 mL of ethanol 80% and shaken in a dark place with magnetic shaker for 72 hours. The extract was filtered twice and lyophilized in oven (55–60°C) for vaporization of water. Then, dry extract was reconstituted in normal saline in a cool and dark place.

### 2.2. Animals and Treatment

Twenty-five adult NMRI male mice with a weight of 30–35 g and age of 6–8 weeks old were purchased from Shiraz Animal Institute (Shiraz, Iran) and kept at the animal house of Fasa University of Medical Sciences (Fasa, Iran). The animals were placed at 12 h light/dark cycle, 22°C, and fed with standard commercial laboratory chew and water. The research was conducted in accordance with the guidelines of National Research Council (affiliated to the Fasa University of Medical Sciences, Fasa, Iran). The animals were randomly divided into five equal groups including (1) control group: mice with normal mode, (2) sham group: mice that intraperitoneally (IP) received physiological saline for 2 weeks, (3) single FA treatment group (FA group): mice that received 10 mg/kg FA (1/10 diluted) as IP for 14 days twice per day (Merck, Darmstadt, Germany) [12], (4) single Fc leaf extracts treatment group (Fc group): mice that received 200 mg/kg Fc extracts for 14 days by oral gavage [31], and (5) both Fc leaf extracts and FA-treated group (Fc + FA group): mice exposed to FA by administration at a dose of 10 mg/kg (twice per day) IP and receiving Fc leaf extracts at the dose of 200 mg/kg via oral gavage for 2 weeks.

### 2.3. Spermatological Studies

#### 2.3.1. Epididymal Sperm Preparation and Sperm Quality Evaluation

Following treatments, mice were sacrificed by cervical dislocation for epididymal sperm preparation. The right cauda epididymides were excised and dissected in 1.5 mL prewarmed phosphate-buffered saline (PBS, pH = 7.4) at 37°C. Gentle agitation of the tissue was applied to enable spermatozoa to disperse (20–25 min). Semen samples were incubated at 37°C for 20–25 min and sperm parameters were analyzed according to criteria of the World Health Organization (fifth edition) with some modifications [38–41] under light microscope at a magnification of ×400.

#### 2.3.2. Assessment of Sperm Motility

The percentage of motile spermatozoa was determined after preparation of sperm suspension by repipetting. A 5 *μ*L drop of the suspension was transferred on a clean glass slide and 15–20-second film was recorded using video camera in five fields from each slide. Sperm motility was assessed via counting progressive, nonprogressive, and immotile spermatozoa after analyzing the recorded films.

#### 2.3.3. Assessment of Sperm Viability

To assess the percentage of viable sperm, we performed 7 *μ*L trypan blue staining by mixing 20 *μ*L of the sperm suspension. The percentage of live (unstained) and dead (blue stained) spermatozoa was recorded under a light microscope at magnification of ×400.

#### 2.3.4. Assessment of Sperm Count

Sperm count was determined by dilution of 1 mL of the sperm suspension with 1 mL of 10% FA fixative. Ten microliters of mixture was transferred into a haemocytometer and sperm count was evaluated per 250 small squares of a haemocytometer.

### 2.4. The Gonadosomatic Index (GSI) Calculation

The body weight of each mouse was recorded and the testes were removed and weighted. The ratio of both testes' weight to the body weight was calculated, and the percentage was determined and recorded as GSI.

### 2.5. Histopathological Studies

The right-side testes from the control and experimental mice were removed and weighed. After fixation of testes with Bouin's fluid at room temperature for 24 h, routine tissue preparation was done. Briefly, the tissues were transferred to 70% alcohol, dehydrated by passing through ascending grades of alcohol, after which the tissues were cleared in xylene and finally embedded in paraffin wax. Using a rotary microtome, 5 *μ*m thickness sections were cut and stained with Haematoxylin-Eosin (H&E) protocol. The stained slides were observed in a research microscope and images were captured.

### 2.6. Statistical Analyses

Data analyses were done with SPSS 16.0 software and are shown as mean ± SD. Statistical analysis included analysis of variance, the Duncan test for multiple comparisons, and the Mann-Whitney analysis. Significance was defined as a *P* value of less than 0.05.

## 3. Results and Discussion

### 3.1. Testicular Weight, Body Weight, and GSI

**Figure 1:**
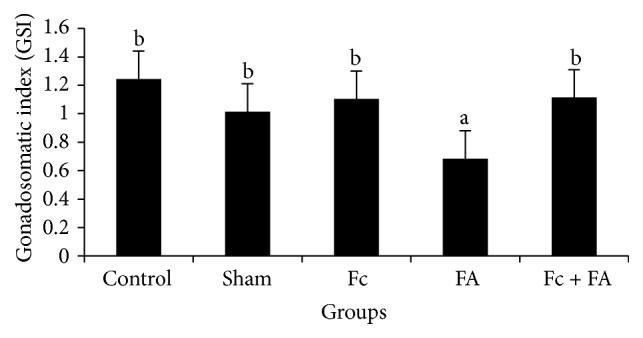
The effect of* Ficus carica* (Fc) leaf extract on the GSI of adult male mice in control, sham, formaldehyde- (FA-) treated, Fc leaf extract-treated, and both FA and Fc leaf extract-treated groups. Homogenous subsets were defined with a to b characters.

The mean ± standard deviation of (testicular and body) weights was (0.23 ± 0.005, 19.1 ± 3.6), (0.239 ± 0.017, 23.7 ± 3.11), (0.227 ± 0.035, 21.2 ± 5.26), (0.133 ± 0.052, 19.9 ± 4.44), and (0.223 ± 0.014, 20.8 ± 4.54) g in the control, sham, Fc, FA, and Fc + FA groups, respectively. The highest amounts of GSI were found in the control (1.24 ± 0.002), Fc (1.1 ± 0.002), Fc + FA (1.11 ± 0.002), and sham (1.01 ± 0.001) groups (*P* = 1), while the lowest amount of those was observed in the FA (0.68 ± 0.002) group (*P* = 0.182). As seen in Figure 1, the GSI of mice in FA-treated group decreased significantly in comparison to those of other groups (*P* ≤ 0.05). This finding is consistent with those of previous reports which found that testicular weight and so GSI were decreased in FA-treated mice [7, 10, 12, 17, 42]. Exposure to FA in the form of inhalation [17, 43] or IP administration [15] can induce some anatomical disturbances in the testes including atrophy of the seminiferous tubules, disorganisation of the seminiferous epithelial cells, and a decrease in the number or degeneration of spermatogenic and Leydig cells [12]. Moreover, several investigators reported the inhibition of spermatogenesis and induction of germ cell apoptosis after FA exposure [11, 15, 18–20] probably by increasing the production of ROS [11, 44] or reduction of metalloenzymes such as zinc and copper which is an important antioxidant enzyme in the cellular protection from ROS [18]. Various studies showed that FA increase the ROS production in many tissues [11, 45, 46] including testes [7] that can inhibit the activity of spermatozoa and increase germ cell apoptosis [11, 14, 47].

In this study, Fc was found to cause an increase in the GSI that may be due to the fact that it contains several antioxidants [27–34].

### 3.2. Spermatological Studies

#### 3.2.1. Sperm Viability

**Figure 2:**
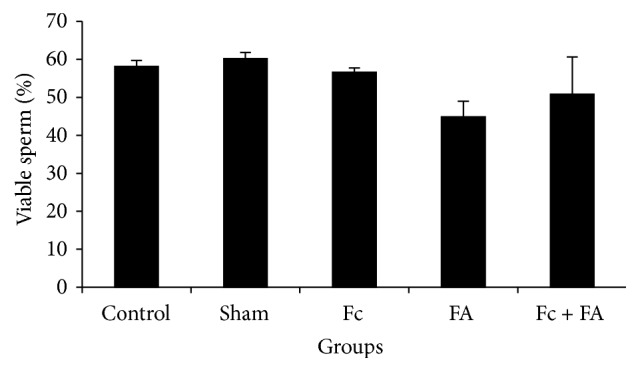
The effect of* Ficus carica* (Fc) leaf extract on the viability of cauda epididymis sperm in adult male mice in control, sham, formaldehyde- (FA-) treated, Fc leaf extract-treated, and both FA and Fc leaf extract-treated groups. There are no homogeneous subsets.

The sperm viability of control mice was about 58.11 ± 1.62 percent, whereas in sham group 60.18 ± 1.6, in Fc group 56.58 ± 1.15, in FA-treated group 44.84 ± 4.17, and in Fc + FA group 50.85 ± 9.78 percent viable sperms were observed. The differences in the viability percent between control and other groups were not significant (*P* > 0.05; Figure 2).

#### 3.2.2. Sperm Count

**Figure 3:**
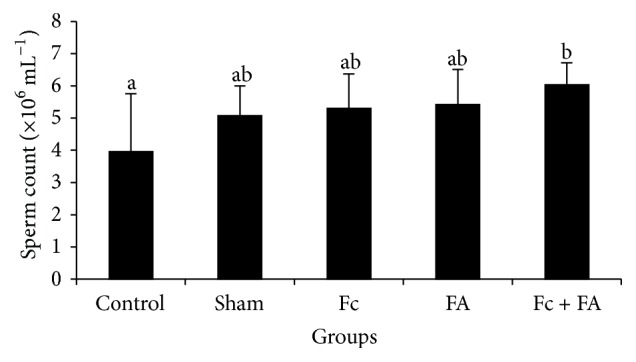
The effect of* Ficus carica* (Fc) leaf extract on the number of cauda epididymis sperm in adult male mice in control, sham, formaldehyde- (FA-) treated, Fc leaf extract- treated, and both FA and Fc leaf extract-treated groups. Homogenous subsets were defined with a to b characters.

The maximum quantities of sperm count were observed in the Fc + FA (60.4 ± 6.87 × 10^6^ sperm/mL), FA (54.2 ± 1.09 × 10^6^ sperm/mL), Fc (53 ± 1.07 × 10^6^ sperm/mL), and sham (50.8 ± 9.28 × 10^6^ sperm/mL) groups (*P* = 0.252) and the minimum of those was seen in the control (39.6 ± 1.8 × 10^6^ sperm/mL), sham, Fc, and FA groups (*P* = 0.086). A significant increase in sperm number was observed in mice given Fc + FA, when compared to the control mice; however, there were no significant changes in other parameters between these two groups (*P* > 0.05; Figure 3).

These results suggest that administration of Fc in FA-treated mice successfully increased the sperm count. This positive effect may be due to the inhibition of lipid peroxidation which occurs by hydroxyl radical scavenging activity of Fc [29]. It seems that Fc can convert the adverse effects of FA through inhibition of oxidative stress and ROS production [29]. ROS were applied by signaling cascade as essential intermediate messenger molecules in the process of apoptosis [48]. Thus, inhibition of ROS production also can have a positive effect on the sperm count and increase in the number of sperm possibility achieved by antiapoptotic effects of Fc [49]. A possible explanation for this might be the presence of antioxidant agents such as phenolic compounds in the Fc extracts that prevent oxidant-induced apoptosis [27, 30, 31]. Antioxidants block the formation of new ROS or act as scavengers and remove ROS already generated [50]. In 2014, Takahashi et al. pointed to some of the components in fig leaves and reported that caffeoylmalic acid (CMA) was the most abundant polyphenol in Fc that exhibited antioxidant activity similar to that of vitamin C or catechin [51]. Several studies have also reported treatment effects of antioxidants, for example, manganese, melatonin, and vitamin E, on the testicular damage and abnormal sperm parameters induced with FA [11, 12, 52]. Another possible explanation for increase in the sperm count in Fc-treated mice may be due to the effects on the concentration of male sexual hormones. On a survey performed on rats, Samsulrizal et al. reported that* Ficus deltoidea* has significant beneficial effects on the testosterone level, and sperm count in diabetic rats [37].

#### 3.2.3. Sperm Motility

**Figure 4:**
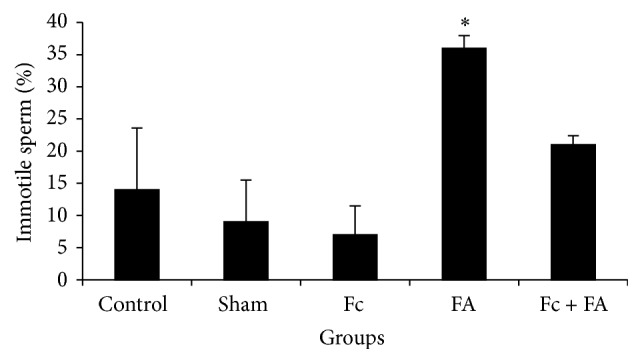
The effect of* Ficus carica* (Fc) leaf extract on the motility of cauda epididymis sperm in adult male mice in control, sham, formaldehyde- (FA-) treated, Fc leaf extract- treated, and both FA and Fc leaf extract-treated groups. *∗*: significant differences with sham and Fc-treated groups.

*(1) Immotile Sperm Percent*. The immotile sperm percent of mice significantly increased in group that received FA (36 ± 1.94) in comparison to sham (9 ± 6.51) and Fc-treated (7 ± 4.47) groups (*P* ≤ 0.05; Figure 4). The percentage of immotile sperm in the control and Fc + FA groups was 14 ± 9.61 and 21 ± 1.43, respectively.

In this study, immotile sperms increased in FA-treated mice. It seems that FA increased the immotile sperms by increase in ROS production that plays an important role in sperm motility. High levels of ROS are associated with lipid peroxidation of the sperm outer membrane that causes loss of motility [53]. It has been suggested that interference in processes of cell membrane ion-exchange and its enzymes decrease sperm motility [54, 55]. A number of studies have found that ROS inhibits intracellular enzyme and so ATP cannot be existing for sperm motility [54, 56, 57]. In 2000 Woo et al. demonstrated that enzymatic activity of Na/K-ATPase, as an ion pump involved in the movement, and eventually the sperm motility reduced following the changes inducing peroxidation of sperm membrane components [54]. Köse et al. exposed rats to FA vapor (10 ppm/1 hour) for 35 days and observed the damaging effects of FA on sperm motility [58]. Tang et al. reported that IP administration of FA at doses of 0.2, 2, and 20 (mg/kg) had negatively impacted on sperm motility in rats [15]. A similar result was observed by Vosoughi et al. that FA vapor increased immotile sperm [16]. According to Mazzilli et al. report, immotile sperm are able to produce anion super oxidase, which is an oxidative factor by itself that can decrease sperm motility [59]. Thus, it seems that decreased sperm motility following FA treatment depends on various factors such as increasing in the production of ROS and shrinking of testis tissue neurogenesis [16, 60, 61].

In this study, FA administration was found to cause an increase in immotile sperm collected from the cauda epididymis. It is an established fact that sperm that go out the testis are not mature physiologically and during their epididymal passage, such maturation happens [62] leading to initiation of motility. This process depends on the modifications that sperm undergo with reference to the small molecular weight compounds and surface proteins [63–66] which are secretory products of the principal cell [65]. The observation that in the FA-treated mice the cauda epididymal immotile sperm increased leads to the speculation that due to a toxic manifestation of FA the principal cells do not secrete such proteins, interpreting the sperm not initiated in motility.

**Figure 5:**
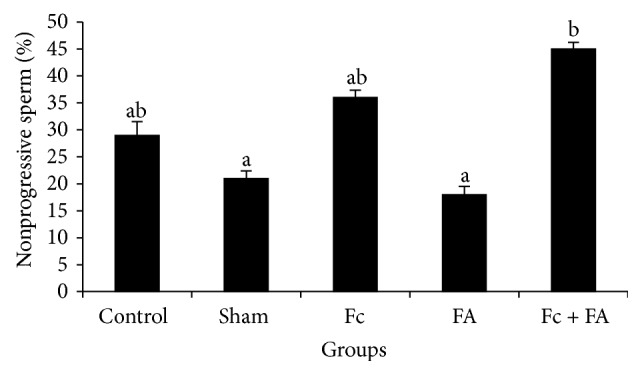
The effect of* Ficus carica* (Fc) leaf extract on the nonprogressive motility of cauda epididymis sperm in adult male mice in control, sham, formaldehyde- (FA-) treated, Fc leaf extract-treated, and both FA and Fc leaf extract-treated groups. Homogenous subsets were defined with a to b characters.

*(2) Nonprogressive Motility.* The nonprogressive sperm percent improved significantly in mice given Fc + FA (45 ± 1.22) compared to the sham (21 ± 1.38) and FA (18 ± 1.48) groups (*P* ≤ 0.05; Figure 5). Groups in the highest homogeneous subset of the nonprogressive sperm included Fc + FA, Fc (36 ± 1.34) and control (29 ± 2.55; *P* = 0.167) and groups in the lowest one contained FA, sham, control, and Fc-treated (*P* = 0.133).

After Fc administration, a significant increase in nonprogressive sperms was seen that may be due to antioxidant effects of Fc. These results were similar to results obtained by Samsulrizal et al. that reported the positive effects of* Ficus deltoidea* leaf extracts on sperm motility in alloxan-induced male diabetic rats [37].

**Figure 6:**
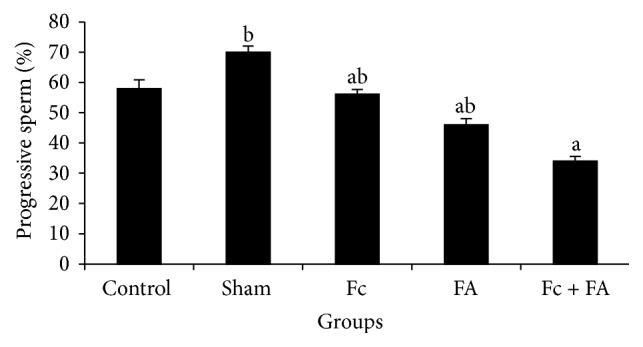
The effect of* Ficus carica* (Fc) leaf extract on the progressive motility of cauda epididymis sperm in adult male mice in control, sham, formaldehyde- (FA-) treated, Fc leaf extract-treated, and both FA and Fc leaf extract-treated groups. Homogenous subsets were defined with a to b characters.

*(3) Progressive Motility*. The lower subset of progressive sperm included the Fc + FA (34 ± 1.51), FA (46 ± 2.07), Fc (56.1 ± 1.52), and control (58 ± 2.86) groups (*P* = 0.104), whereas the upper subset comprised the sham (70 ± 2), control, Fc, and FA groups (*P* = 0.104). The percent of progressive sperm in Fc + FA group decreased significantly in comparison to those of the sham group (*P* ≤ 0.05; Figure 6). Based on these results, no adverse effect of FA was seen in progressive sperms. These findings further support the idea of Vosoughi et al. who claimed that FA could be having the adverse effects on sperm progressive motility in two specified time points, one day and 35 days after exposure [16]. The period of the full cycle of epithelial cells regeneration in mice seminiferous tubules is 8.6 days, whereas for spermatogenesis it is 35 days. Therefore, changes in physiological parameters of sperm are evaluated better after a period of 35 days [67, 68].

### 3.3. Histopathological Changes in the Testis

**Figure 7:**
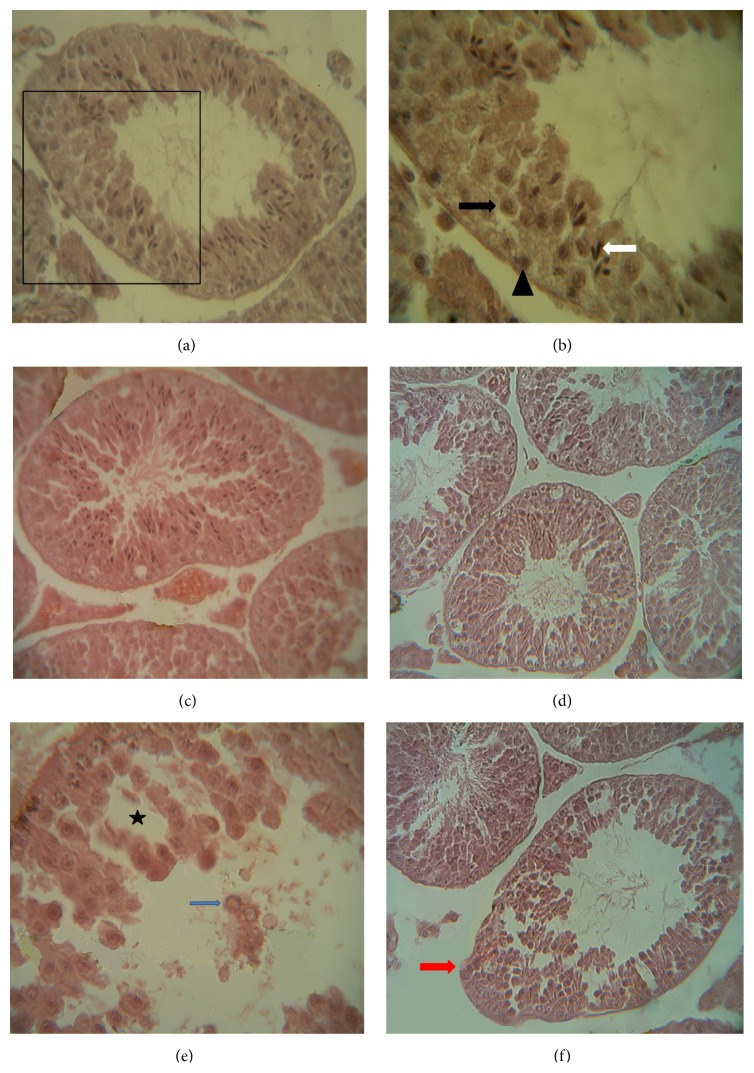
Morphology of mouse seminiferous tubules in the testis of (a) control, (c) sham, and (d) Fc-treated groups showing normal histoarchitecture of seminiferous tubules in the Haematoxylin and Eosin (H&E) stained paraffin sections. (b) Further magnification of the selected part of (a) showing normal spermatogenic cell line including spermatogonia (arrowhead), primary spermatocyte (black arrow), and late spermatid (white arrow). (e) FA-treated group showing disorganized and vacuolated seminiferous epithelium (asterisk), spermatogenic arrest, and lumen filled with immature germ cells (blue arrow). (f) In the Fc + FA-treated group, seminiferous tubule with spermatogenic arrest (red arrow) was rarely observed. (a, c, d, f) ×400 and (b, e) ×1000 magnification.

Light microscopy studies of seminiferous tubules of mice in the control, sham, and Fc-treated group showed the organized seminiferous tubules with tall seminiferous epithelium, narrow lumen, and compact arrangement with the interstitium. The seminiferous tubules of the above mentioned groups showed different stages of the spermatogenic cycles (Figures 7(a)–7(d)).

Histological examinations of the testes showed significant histological changes in mice testes in the FA-treated group. In this group, the seminiferous epithelium was disorganized and the epithelium of seminiferous tubules appeared highly vacuolated and the shape of these vacuoles designates death or exfoliation of clones of spermatogenic cell lines. Some seminiferous tubules with arrest spermatogenic cycle were also observed. Detached germ cells or degenerating germinal elements were filled in the lumen (Figure 7(e)).

In the Fc + FA-treated group, seminiferous tubules with spermatogenic cycle arrest were hardly ever seen among the seminiferous tubules with normal spermatogenic cycles (Figure 7(f)).

In the present study, it was observed that administration of FA led to alteration in both the histoarchitecture of testis and irregular spermatogenesis. The pathological changes observed in seminiferous tubules of the FA-treated testis are consistent with those of previous reports which found that FA exposure can induce several testes anatomical disturbances such as disorganisation of the seminiferous epithelial cells [12, 15, 17, 43] and the inhibition of spermatogenesis [11, 15, 18–20]. These histopathological changes in the seminiferous tubules and spermatogenesis process have been explained by the different mechanisms. One of these is cytotoxic effect of FA, according to the Ma and Harris study [69]. Another mechanism was reported by Feldman [70], who showed that FA administration caused the arrest of nucleic acid synthesis and proteins and it can also increase the production of ROS in many tissues which are important mediators of cellular injury and oxidative damage [11, 45, 46]. Surveys such as that conducted by Zhou et al. have shown that FA decrease the effectiveness of the rat testicular antioxidant system and increase the testicular lipid peroxidation [11]. One of the most important products of lipid peroxidation is malondialdehyde that interferes with protein biosynthesis by forming adducts with DNA, RAN, and protein [71]. Oxidative stress is an important mechanism of testicular damage [11]. In another investigation, Golalipour et al. showed that FA altered levels of trace elements, including zinc, copper, and iron in the rat testicular tissue [17] that is the other possible mechanism in oxidative stress, either by production of a complex with soluble cellular chelating agents such as ADP or directly by oxidative stress, and can have an adverse effect on spermatogenesis [72, 73].

One of the most prominent changes occurring in the epithelium of the seminiferous tubules on treatment with FA was extensive vacuolation. It seems that this process is due to premature exfoliation of the spermatogenic cells in the adluminal compartment leading to development of large spaces in the seminiferous epithelium [74]. It is further speculated that FA causes disturbance to spermatogenesis through affecting the junctional complexes. This is in agreement with Arican's (2009) findings which reported the effects of FA on the complete impairment of intercellular junctional complexes and disturbance of the tissue integrity in nasal mucosa [8]. Thus, it could be assumed that the disturbance to the junctional complexes can also be the basis for the premature exfoliation of the germinal cells.

In this study, testes histological examinations of the Fc + FA group revealed that administration of Fc leaf extracts prevented from some damage effects of FA. These preventive effects can be related to the presence of several antioxidants in Fc leaf extracts that had been stated previously [27–34].

## 4. Conclusion

The findings of this study showed that FA adversely affects cauda epididymis sperm parameters of mice, including increased percentages of immotile sperm and decreased GSI. Also, disorganized and vacuolated seminiferous epithelium, spermatogenic arrest, and lumen filled with immature germ cells were observed in the seminiferous tubules of FA-treated mice. Meanwhile, Fc increased GSI, sperm count, and nonprogressive motility of sperms in mice treated with FA. Furthermore, seminiferous tubule with spermatogenic arrest was rarely seen in this group. Although the exact mechanism of action was unknown, these positive effects may be due to the antioxidant effects of Fc. Therefore, it can be used for male infertility treatment caused by FA exposure.
